# Ferroelectric Smectic Liquid Crystalline Materials with Different Degree of Chirality

**DOI:** 10.3390/ma18102343

**Published:** 2025-05-17

**Authors:** Michał Czerwiński, Mateusz Filipow, Klaudia Łuczak, Dorota Węgłowska

**Affiliations:** 1 Institute of Chemistry, Military University of Technology, Kaliskiego 2, 00-908 Warsaw, Poland; mateusz.filipow@wat.edu.pl (M.F.); dorota.weglowska@wat.edu.pl (D.W.); 2 Institute of Organic Chemistry, Polish Academy of Sciences, Kasprzaka 44/52, 01-224 Warsaw, Poland; klaudia.luczak@icho.edu.pl

**Keywords:** ferroelectricity, liquid crystals, electro-optical effects, physicochemistry, self-assembly materials

## Abstract

Ferroelectric liquid crystals (FLCs) are key materials for high-speed electro-optical applications, yet achieving optimal properties over a broad temperature range down below room temperature remains a challenge. This study presents a novel series of systematically designed FLC mixtures, incorporating components with three degrees of chirality—achiral systems, with one center of chirality and with two centers of chirality—to optimize the mesomorphic stability, electro-optical response, and physicochemical properties. The strategic doping by chiral components up to a 0.2 weight fraction extends the temperature range of the ferroelectric phase while lowering the melting temperature. Notably, mixtures containing two chiral centers exhibit shorter helical pitches, while increasing chirality enhances the tilt angle of the director and spontaneous polarization. However, in a mixture containing all three types of chirality (CchM), spontaneous polarization decreases due to opposing vector contributions. Switching time analysis reveals that a system with achiral components and those with two centers of chirality (A-BchM) exhibits the fastest response, while CchM demonstrates only intermediary behavior, caused by its high rotational viscosity. Among all formulations, those containing compounds with two centers of chirality display the most favorable balance of functional properties for deformed helix ferroelectric liquid crystal (DHFLC) applications. One such mixture achieves the lowest melting temperature reported for DHFLC-compatible FLCs, enabling operation at sub-zero temperatures. These findings pave the way for next-generation electro-optical devices with enhanced performance and appropriate environmental stability.

## 1. Introduction

Liquid crystal (LC) materials, particularly those exhibiting smectic phases, are crucial for advanced electro-optical applications, including high-performance displays, spatial light modulators, and optical switches [1,2,3,4,5,6,7,8,9]. Liquid crystals exist in a mesomorphic state and may comprise multiple sub-phases, each distinguished by its specific molecular ordering. Among these, nematic liquid crystals (NLCs) exhibit long-range orientational order without positional order, meaning their molecular long axes align along a common director (*n*) but lack a fixed spatial arrangement. In contrast, smectic liquid crystals (SLCs) are organized into layered structures, maintaining orientational order while forming distinct, structured arrangements. Among these, the SmA and SmC phases are particularly significant due to their widespread applicability. In the SmA phase, molecules, i.e., the director, align perpendicularly to the layers, whereas in the SmC phase, the director tilts at an angle (*θ*) with respect to the layer normal.

A crucial subclass of the smectic phases is the chiral smectic C (SmC*) phase [10], which, due to its intrinsic chirality, exhibits spontaneous polarization (*P_s_*), rendering it ferroelectric. This characteristic makes the SmC* materials—commonly referred to as ferroelectric liquid crystals (FLCs)—highly desirable for applications requiring high-speed electro-optical switching [11]. However, it is still almost impossible to reach the desirable properties of the FLCs in a single molecule material [12,13]. That is why the design of binary and multicomponent FLC mixtures remains an effective tool for tuning their properties and getting the required ones by the specific applications [13,14,15].

**Figure 1:**
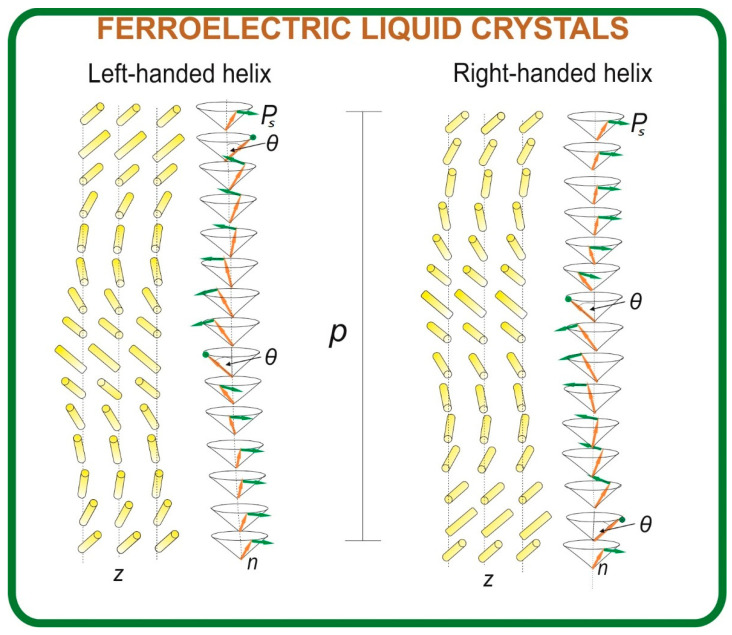
Schematic illustration of molecular orientation leading to the formation of a heliconical structure in FLCs; it is characterized by helical pitch (*p*) and distinct handedness. The figure also highlights the molecular director (*n*), helix axis (*z*), tilt angle of the director (*θ*), and spontaneous polarization vector (*P_s_*).

The electro-optical performance of FLCs depends on several critical parameters, including the mentioned tilt angle (*θ*) and spontaneous polarization (*P_s_*), as well as the helical pitch length (*p*), switching time (*τ*), and rotational viscosity (*γ_φ_*). The helical pitch represents the periodicity over which molecular orientation, i.e., the director, rotates for 360°, forming a helical superstructure (see Figure 1). The switching time *τ* defines the response speed between two stable electro-optical states, while the rotational viscosity determines the ease of molecular reorientation under an external electric field. Achieving materials with the SmC* phase and optimal properties over a broad temperature range remains a key challenge in FLC development [14,16,17,18,19,20,21,22,23,24,25,26,27,28].

There are two principally different electro-optical effects exploiting FLCs: the surface-stabilized ferroelectric liquid crystal (SSFLC) effect [10] and the deformed helix ferroelectric liquid crystal (DHFLC) effect [29,30]. The SSFLC effect is based on surface-induced synclinic ordering, where interactions with confining substrates unwind the intrinsic FLC helical structure. This requires ultra-thin electro-optical cells with thicknesses below the helical pitch. The molecular alignment changes under an applied electric field, stabilizing in two synclinic states even after the field is removed. This bistability enables high-contrast optical switching, making SSFLCs ideal for memory-based electro-optical devices.

In contrast, the DHFLC effect leverages the deformation of the helical structure under an electric field. In this configuration, the helix axis is aligned parallel to the electro-optical cell, defining the optical axis of the medium. Unlike SSFLCs, the heliconical structure remains intact, requiring materials with a short helical pitch (below the cell thickness). The application of a low-voltage electric field deforms the helix, inducing a tilt in the optical axis and modulating light polarization. At small voltages, the tilt angle varies linearly with field intensity, whereas at higher voltages, the helix unwinds, yielding SSFLC-like behavior. While DHFLCs lack bistability, they offer grayscale rendering, operate at lower driving voltages, and require no threshold voltage, making them promising candidates for next-generation photonic devices, including optical sensors and fast-switching display technologies [31,32].

In our days, the FLC mixtures are formulated using two primary strategies: (i) by introducing a chiral dopant—either with one or two stereocenters—into a low-viscosity achiral SmC base mixture [33,34,35] or (ii) by employing FLC compounds exclusively with one center of chirality [18,36,37]. The former approach allows the fine-tuning of material properties but often results in a lower tilt angle of the director. Conversely, FLC materials based on compounds with one center of chirality typically exhibit high tilt angles but present challenges in optimizing other physicochemical parameters, often leading to increased viscosity [38]. Both approaches share a common drawback: difficulty in achieving low melting temperatures, which are necessary for stable FLC operation in both DHFLC and SSFLC modes at sub-zero temperatures. The ability to develop FLC materials that retain their electro-optical performance at low temperatures is essential for applications requiring high stability in extreme environmental conditions (e.g., low temperature).

**Figure 2:**
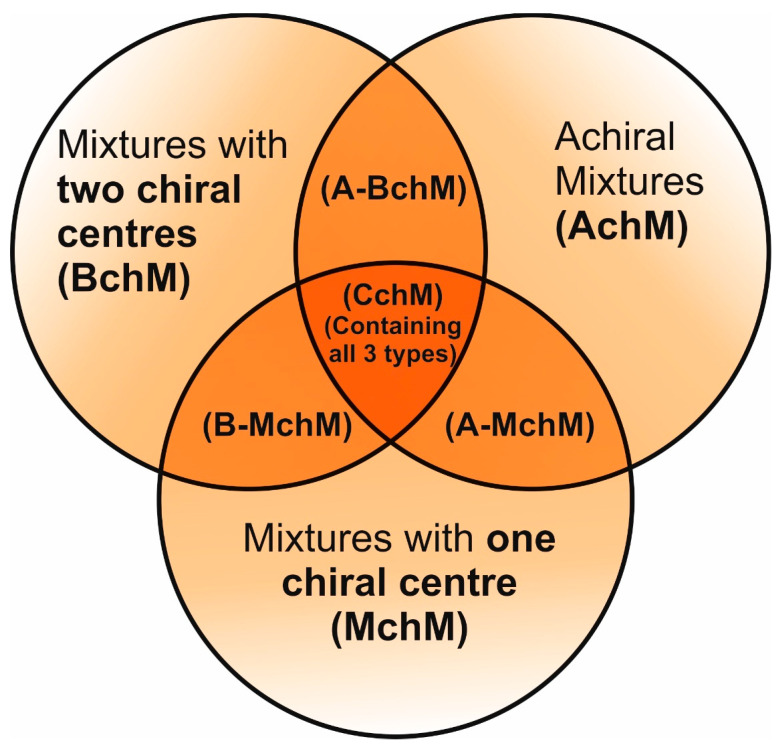
A schematic representation illustrating the design concept for the studied FLC mixtures based on compounds with different degrees of chirality.

Here, we present a novel series of systematically designed FLC mixtures built up in a way to elucidate the impact of chirality on mesomorphic stability, the electro-optical response, and key physicochemical parameters (Figure 2). For the first time, we have developed a complex chiral mixture (CchM) incorporating compounds lacking chirality, with a single chiral center and with dual chiral centers (CchMs). This approach has enabled the formulation of an FLC material with an exceptionally low melting temperature but with the preservation of other physicochemical properties, making it highly suitable for DHFLC applications. These findings represent a significant advancement in the development of next-generation ferroelectric LC materials, offering new avenues for their integration into cutting-edge electro-optical technologies.

## 2. Materials and Methods

### 2.1. FLC Mixtures

In this study, liquid crystal materials were systematically developed based on three primary base mixtures. The initial step involved determining the eutectic composition of each base mixture using a MATLAB (Version 5.3.1)-based computational program developed at the Military University of Technology (MUT). This program employs thermodynamic modeling based on Equations (1) and (2), as follows:

(1) $$lnX_{k}=-\frac{∆H_{m}^{k}}{R}\left(\frac{1}{T}-\frac{1}{T_{m}^{k}}\right)$$

(2) $$\sum_{k=1}^{n}X_{k}=1$$

where $∆H_{m}^{k}$ and $T_{m}^{k}$ denote the enthalpy of fusion and melting temperature of component *k*, respectively. The variable $X_{k}$ represents the mole fraction of component *k*, *n* is the total number of components, *R* is the universal gas constant, and *k* serves as the component index.

The first developed base mixture, referred to as the eutectic achiral mixture (AchM), comprises ten two-ring organic compounds in varying specific proportions [34]. These compounds share a characteristic core structure typical of the SmC materials, consisting of a pyrimidine and a benzene ring. However, they differ in their terminal groups—either aliphatic or alkoxy in various configurations—and in the length of these groups, which range from four to ten carbon atoms. The detailed composition of the AchM mixture, along with the weight fraction values of its constituents, is presented in Table 1.

The second base mixture, referred to as the eutectic MchM mixture, is a binary system composed of compounds featuring a single chiral center [38]. Both constituents exhibit a three-ring rigid core with terminal alkoxy substituents. For both compounds, hydrogen atoms within the unbranched alkyl chain are partially substituted with fluorine atoms, and an ester group is positioned between two benzene rings. A key structural distinction lies in the lateral substitution on the benzene ring close to the chiral chain: one compound incorporates a chlorine atom, whereas the other one possesses a fluorine atom. The detailed composition of the MchM mixture, including the relative proportions of its constituents, is presented in Table 2.

The third base mixture, designated as the BchM mixture, consists of compounds possessing two chiral centers [39]. These molecules are characterized by a rigid core comprising three benzene rings and two identical terminal chiral chains containing ester groups. The primary structural differentiation between the two constituents arises from the presence or absence of a nitrogen atom in the central benzene ring. Specifically, one compound consists solely of benzene rings, while in the other, a pyridine ring replaces one of the benzene rings. The composition of the BchM mixture is presented in Table 3.

Initial characterization was performed on the base mixtures themselves. Subsequently, novel formulations were obtained by combining these base mixtures in various weight ratios, followed by further experimental investigations of the resulting compositions. These additional systems, designed by mixing the three base mixtures in different proportions, are summarized in Table 4.

### 2.2. Polarizing Optical Microscopy and Differential Scanning Calorimetry

To measure phase transition temperatures and identify phases, a polarizing optical microscope (POM) was used. The sample was placed between crossed polarizers and analyzed on a heating stage that controlled its temperature, allowing for the determination of phase transition temperatures. Both heating and cooling were performed at a rate of 2 °C/min, and the apparatus provided temperature measurements with an accuracy of 0.1 °C. The experiments were conducted using an Olympus BX51 POM (Tokyo, Japan) equipped with a LINKAM THMS 600 heating stage (Redhill, UK). The microscope image was observed on a monitor via an FW ColorView III camera (Olympus, Tokyo, Japan), while the heating stage temperature was controlled using a Linkam CI-94 temperature controller (Redhill, UK). To further investigate thermal properties, differential scanning calorimetry (DSC) was used to determine the phase transition temperatures and enthalpy changes. During the experiments, the samples were heated and cooled at a rate of 2 °C/min, while the measurement chamber was flushed with gaseous nitrogen at a flow rate of 20 mL/min. The DSC 204 F1 calorimeter from NETZSCH (Selb, Germany) was used for these studies. The weight of the samples used for the DSC measurements was 10.00–12.00 mg.

### 2.3. Helical Pitch Measurement

For the measurement of the helical pitch length (*p*), the studied material was applied onto a glass slide coated with a 5% solution of octadecyltrimethylammonium bromide (C_18_H_37_(CH_3_)_3_N⁺Br^−^) in ethanol, which ensures the homeotropic alignment. After the solvent evaporated, the test material was deposited, and the sample was heated by a heating stage until it transitioned into the isotropic (Iso) liquid phase. The prepared slide was then placed into a brass cuvette, and the measurement was initiated. First, a reference curve was always recorded without the test material, followed by the actual measurement with the sample applied. The highest temperature used was the transition temperature from the lower-ordered phase to the chiral tilted phase, while the lowest achievable temperature was 2 °C. The cooling was performed at a rate of 5 °C/min, with measurements taken at 3 °C intervals. Each measurement was preceded by a temperature stabilization period of 4 min. Helical pitch determination was based on the selective reflection phenomenon [38] and analyzed using a SHIMADZU UV-3600 UV-VIS-NIR spectrophotometer (Kyoto, Japan), operating within the 360–3000 nm wavelength range. Temperature control was achieved using the U7 MLW thermostat (SHIMADZU, Kyoto, Japan). The selectively reflected wavelength (*λ_s_*) was determined at half the height and width of the reflection band, and the helical pitch was subsequently calculated using Equation (3), as follows:

*p* = *λ_s_*/*n_av_* (3)

where *n_av_* is the value of the average refractive index; *n_av_* = 1.5 was taken for calculation [40].

### 2.4. Electro-Optical Properties, Spontaneous Polarization, and Tilt Angle

To investigate the electro-optical properties, a test cell consisting of two glass plates separated by a 1.6 μm gap was constructed. The plates were pre-coated with a polyimide (Nylon 6) alignment layer and transparent Indium Tin Oxide (ITO) electrodes. The inner surfaces of the cell were rubbed in opposite directions, creating an anti-parallel rubbing alignment. In the isotropic phase, the tested materials were filled into the cell; due to low viscosity and capillary forces, the LC substance was homogeneously distributed throughout the entire volume of the test cell. The prepared cell was then placed on a Linkam HFS91 heating stage (Redhill, UK) in a BIOLAR PZO polarizing microscope (Polskie Zakłady Optyczne, Warsaw, Poland) equipped with a THORLABS PDA100A-EC photodetector (Newton, NJ, USA). Electro-optical measurements were conducted using a Rohde & Schwarz HMF2550 arbitrary function generator (Munich, Germany), an HM0724 oscilloscope (Rohde & Schwarz, Munich, Germany), and a Linkam TMS93 temperature controller (Redhill, UK), with a twenty-fold amplification of the control signal using an FLC F20AD amplifier (Partille, Sweden).

The switching time was determined by measuring the interval at which transmittance reached 90% of its maximum during the transition between two synclinic states, induced by a rectangular electrical signal. The director tilt angle was evaluated by applying a rectangular electrical signal with a voltage range of 14–30 V and a frequency of 30 Hz to the electro-optical cell [41]. This resulted in two synclinic states corresponding to the opposite polarity of the electric field. By rotating the microscope stage, two minima in transmittance were identified for positive and negative applied voltages. These dips, observed when the optical axis and polarization plane were parallel, enabled the determination of the director tilt angle (2*θ*) as the values between the two dark states. Measurements were conducted as a function of temperature, with steps of 2 °C, 3 °C, and finally 5 °C at lower temperatures. Spontaneous polarization measurements were performed using the same electro-optical cell, connected in series with an MCP BXR-07 decade resistor (Transfer Multisort Elektronik, Łódź, Poland) with a resistance of *R*_0_ = 13 kΩ. A triangular voltage of 20–30 V at a frequency of 50 Hz was applied, and measurements were based on the voltage drop across the resistor [42]. Data acquisition was carried out using a custom-developed computer program created at the Military University of Technology in Warsaw in the LabVIEW environment. The active area of the cell was 0.25 cm^2^; 1.6 μm thick cells were used for the measurements.

The temperature dependence of the rotational viscosity (*γ_φ_*) for the selected materials was determined based on the switching time and spontaneous polarization measurements, using the semi-empirical Formula (4) [43], as follows:

(4) $$\;{\;\gamma }_{\varphi }=\frac{1}{1.8}\;P_{S}E\tau$$

where *E = U/d*, *U* represents the applied voltage, and *d* denotes the cell gap.

## 3. Results and Discussion

### 3.1. Mesomorphic Behavior

**Figure 3:**
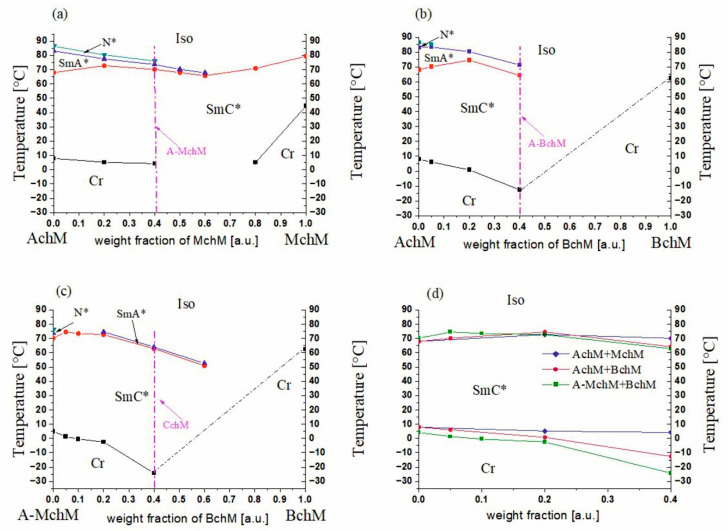
Phase diagrams (data from DSC) for systems: AChM-MChM (**a**), AchM-BchM (**b**), A-MChM-BchM (**c**), and collected phase diagram with melting points and upper-temperature range of the SmC* phase (**d**). The compositions of mixture A-MChM, A-BChM, and CChM are marked with a purple arrow and a dashed line in each phase diagram. “Cr” stands for the crystal phase.

The investigated multicomponent systems consisted of three types of mixtures: (i) achiral (AchM) combined with a mixture of compounds with one chiral center (MchM), (ii) achiral (AchM) combined with a mixture of compounds with two chiral centers (BchM), and (iii) a mixture of compounds without and with one center of chirality (A-MchM) combined with a mixture of compounds with two centers of chirality (BchM), as was presented in Figure 3 and Table 4. The phase diagrams of these systems are shown in Figure 3 and the detailed temperatures and enthalpies of each prepared mixture are collected in Table S1 (ESI).

The achiral AchM mixture exhibits three distinct liquid crystal phases: a broad temperature range tilted SmC phase, the orthogonal SmA phase, and the N phase (see Table S1, ESI). The phase diagram of the AchM-MChM system (Figure 3a) reveals that all its mixtures retain a wide temperature range ferroelectric phase and exhibit melting points below room temperature. Additionally, in mixtures containing up to a 0.6 weight fraction of MchM in AchM, the SmA* phase persists, while the N* phase is maintained up to a composition of 0.4. The mixture consisting of a 0.4 weight fraction of MchM in AchM exhibited one of the lowest melting temperatures and a reasonably high SmC*–SmA* phase transition temperature (above 70 °C), making it the optimal candidate for further studies. This mixture was subsequently designated as A-MchM and served as the basis for preparing the final system, which incorporated components with all three degrees of chirality. In the second system, the AchM mixture was doped with a mixture of compounds with two centers of chirality (BchM) mixture, which has a melting point of 62.4 °C (see Table S1, ESI). The phase diagram of this system (Figure 3b) shows the disappearance of the N* phase at chiral mixture concentrations exceeding a weight fraction of 0.05. Up to a composition of 0.4, the SmA* phase remains present; however, it becomes destabilized in favour of the ferroelectric phase. The temperature range of the SmC* phase expands up to a chiral mixture concentration of 0.2, beyond which it undergoes significant destabilization. The melting temperature decreases substantially, reaching −12.6 °C for the mixture containing a 0.4 weight fraction of BChM. The final system was obtained by combining a mixture of compounds with none, and one center of chirality (A-MchM) with the mixture of compounds with two centers of chirality (BchM). In this system, an increasing concentration of BchM resulted in a further expansion of the SmC* phase while significantly lowering the melting temperature (Figure 3c).

Among the multiple mixtures initially synthesized and analyzed, only three FLC materials were selected for further investigation based on a comprehensive assessment of their mesomorphic properties. This selection process identified three key formulations for advanced studies: (i) the optimized system containing achiral compounds and those with only one center of chirality, 0.6 AchM + 0.4 MchM (A-MchM), (ii) the system with achiral compounds and those with only two centers of chirality 0.6 AchM + 0.4 BchM (A-BchM), and (iii) the optimized system containing all three types of chirality referred to as a complex chiral mixture (CchM). The remaining mixtures from the three examined systems were excluded from further analysis due to either limited phase stability or elevated melting temperatures (Figure 3d), which restricted their suitability for advanced electro-optic applications.

Two of the selected mixtures (A-BchM and CchM) exhibit melting temperatures below 0 °C, while all three maintain a stable ferroelectric phase up to 62 °C (Table 5). Notably, a mixture containing the three types of compounds (achiral, with one chiral center and with two chiral centers) demonstrates the most pronounced extension of the ferroelectric phase range at lower temperatures. DSC traces for A-MchM, A-BchM, and CchM are presented in Figure S1 (ESI).

### 3.2. Helical Pitch

**Figure 4:**
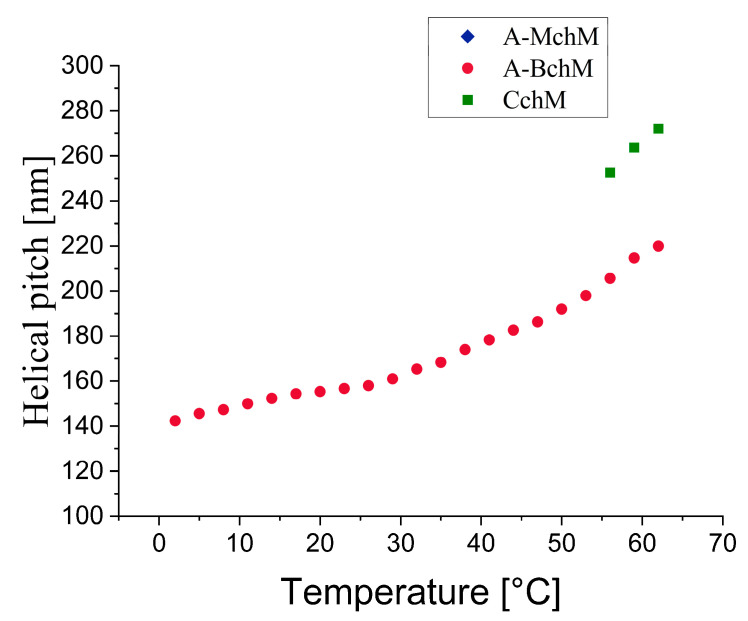
Temperature dependence of the helical pitch length for all tested mixtures as indicated.

Figure 4 shows the temperature dependence of the helical pitch for the selected mixtures. It can be observed that as the temperature decreases, the helical pitch of the A-BchM and CchM mixtures also decreases. Notably, in the A-BchM mixture, the helical pitch remains the smallest across the entire temperature range of the ferroelectric phase. For the other mixtures, the data were only partially recorded due to the limitations of the spectrophotometer’s measurement range. In the case of the CchM mixture, three data points were obtained at temperatures above 55 °C, while at lower temperatures, the selectively reflected wavelength values fell below 360 nm. Conversely, for the A-MchM mixture, the helical pitch values exceeded the upper measurement limit of the spectrophotometer (*p* > 2.0 µm). The exceptionally low helical pitch values recorded for the A-BchM mixture were made possible by the appearance of weak full-pitch bands in the spectra (see Figure S2, ESI). When all three base mixtures are combined, the helical pitch does not further decrease beyond that observed in the A-BchM system. This suggests differential twisting efficiencies between the chiral components, ultimately constraining further helical pitch reduction.

### 3.3. Tilt Angle and Spontaneous Polarization

**Figure 5:**
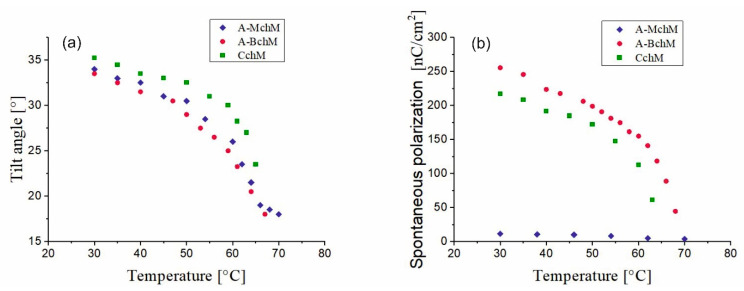
Temperature dependence of the tilt angle (**a**) and the spontaneous polarization (**b**) for mixtures: A-MchM, A-BchM, and CchM.

As shown in Figure 5a, the tilt angle decreases with increasing temperature for all selected mixtures. The highest values of the tilt angle are observed for the CchM mixture, which incorporates all three types of compounds: achiral, with on center of chirality and with two centers of chirality. The other mixtures exhibit quite comparable tilt angle values. Notably, for all selected mixtures, the tilt angle values remain above 32° at 30 °C. The highest values of the spontaneous polarization are observed for the mixture A-BchM (Figure 5b). Introducing a mixture containing compounds with a single chiral center (MchM) into this system results in a reduction in spontaneous polarization. This effect may arise due to the opposing signs of the spontaneous polarization vector generated by the components of BchM and AchM mixtures. The lowest spontaneous polarization values are recorded for mixture A-MchM.

### 3.4. Switching Time and Rotational Viscosity

**Figure 6:**
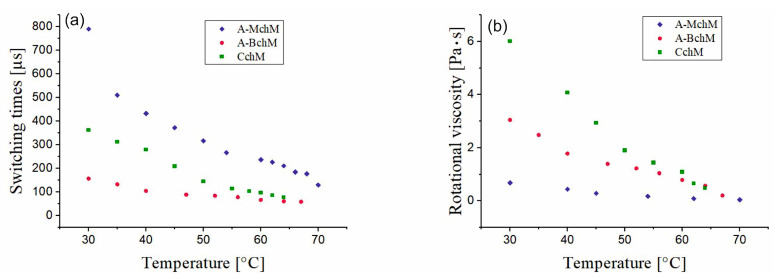
Temperature dependence of the switching time (**a**) and rotational viscosity (**b**) for mixtures: A-MchM, A-BchM, and CchM.

The switching times between the two synclinic states increase with decreasing temperature for all selected mixtures; the most pronounced effect was observed for mixture A-MchM and the lowest one for mixture A-BchM (Figure 6a). Among the examined systems, mixture A-BchM exhibits the shortest switching times across the entire temperature range, while mixture A-MchM shows the longest ones. Interestingly, the CchM mixture demonstrates an intermediate switching behavior, being slower than the A-BchM mixture but faster than the A-MchM mixture. Given that CchM also exhibits the highest rotational viscosity among the three selected mixtures (Figure 6b), this finding suggests a complex interplay between viscosity and spontaneous polarization in the studied systems.

## 4. Conclusions

A systematic approach was employed to design and characterize new multicomponent FLC materials by leveraging three primary eutectic base formulations: mixture of achiral compounds (AchM), mixture of compounds with one center of chirality (MchM), and mixture of compounds with two centers of chirality (BchM). Subsequently, binary and ternary LC systems were developed by combining these base mixtures in a controlled composition, followed by comprehensive investigations into their mesomorphic behavior. As a result, the three best multicomponent mixtures were selected, and their mesomorphic and electro-optical characteristics were investigated.

The findings reveal several critical trends in the mesomorphic behavior of studied LC systems. First, the introduction of chiral components—up to a 0.2 weight fraction—consistently expands the temperature range of the ferroelectric phase and simultaneously lowers the melting temperature across all developed mixtures. The mixture composed of compounds with two chiral centers exhibits a significantly shorter helical pitch compared to those containing one chiral center. The mixture incorporating compounds with three different degrees of chirality exhibits significantly higher tilt angles compared containing only one or only two stereocenters. A similar tendency is observed for spontaneous polarization behavior, which generally increases with chiral content. The longest switching times are observed in systems composed of achiral compounds and those containing only a single chiral center, which can be attributed to their higher viscosity and lower spontaneous polarization. In contrast, A-BchM mixture exhibits the shortest switching times due to the significantly enhanced spontaneous polarization.

The comprehensive results presented in Table 6 indicate that the two mixtures incorporating compounds with two centers of chirality are the most promising candidates for applications utilizing the DHFLC effect. Particularly noteworthy is the mixture containing all three degrees of chirality, as it possesses the lowest reported melting temperature among FLC mixtures developed for the DHFLC effect; much lower than the top-modern NFLC-4-eut mixture [44]. This feature extends the operational range of the ferroelectric phase to significantly lower temperatures, enabling DHFLC functionality well below 0 °C—a capability critical for next-generation low-temperature photonic devices. The primary limitation of this system is its relatively high rotational viscosity, which may contribute to slower switching times, but is still faster than electro-optical system-based nematic LCs. It is worth noting that the studied mixtures exhibit thermodynamic stability, as evidenced by the consistent values of their measured properties even several months after the initial characterization.

These findings establish a robust framework for further design of new liquid crystalline materials with precisely predicted ferroelectric properties, thereby opening new avenues for fast-switching photonic devices and adaptive optical systems, particularly those operating at low temperatures.

## Figures and Tables

**Table 1:** Composition of the base achiral mixture (AchM) [34].

Numbers of Component	Chemical Formulae	Weight Fraction
**1**	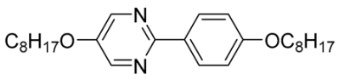	0.16
**2**	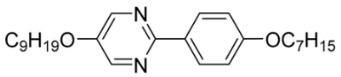	0.01
**3**	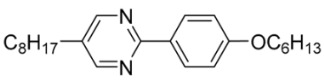	0.02
**4**	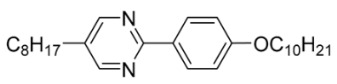	0.05
**5**	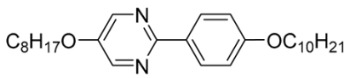	0.19
**6**	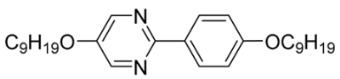	0.14
**7**	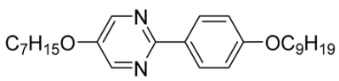	0.05
**8**	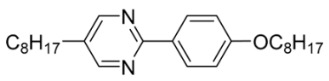	0.13
**9**	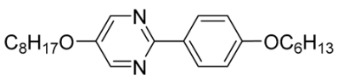	0.03
**10**	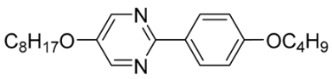	0.22

**Table 2:** Composition of the base MchM mixture, based on compounds with a single chiral center [38].

Numbers of Component	Chemical Formulae	Weight Fraction
**11**	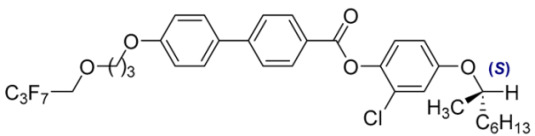	0.52
**12**	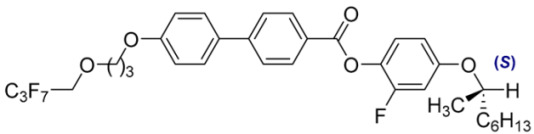	0.48

**Table 3:** Composition of the base BchM mixture, based on compounds with two chiral centers [39].

Numbers of Component	Chemical Formulae	Weight Fraction
**13**	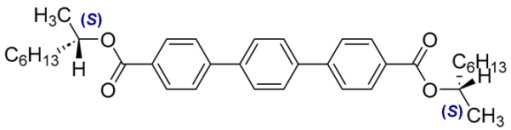	0.44
**14**	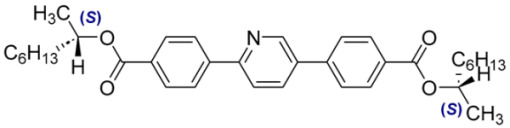	0.56

**Table 4:** Designations and compositions of the developed mixtures incorporating compounds with varying degrees of chirality, categorized within three primary systems: MchM–AchM, AchM–BchM, and CchM–BchM.

	Composition of the Mixture (Weight Fraction)		
Mixture Acronym	AchM Mixture	MchM Mixture	BchM Mixture
0.2 MchM + 0.8 AchM	0.80	0.20	-
0.4 MchM + 0.6 AchM **(designated as A-MchM)**	0.60	0.40	-
0.5 MchM + 0.5 AchM	0.50	0.50	-
0.6 MchM + 0.4 AchM	0.40	0.60	-
0.8 MchM + 0.2 AchM	0.20	0.80	-
0.05 BchM **+** 0.95 AchM	0.95	-	0.05
0.2 BchM + 0.8 AchM	0.80	-	0.20
0.4BchM **+** 0.6 AchM **(designated as A-BchM)**	0.60	-	0.40
0.4 A-MchM + 0.6 BchM	0.24	0.16	0.60
0.6 A-MchM + 0.4 BchM **(designated as CchM)**	0.36	0.24	0.40
0.8 A-MchM + 0.2 BchM	0.48	0.32	0.20
0.9 A-MchM + 0.1 BchM	0.54	0.36	0.10
0.95 A-MchM + 0.05 BchM	0.57	0.38	0.05

**Table 5:** The phase transition temperatures [°C] from DSC (first row) and corresponding enthalpy changes [kJ mol^−1^] (second row), in italic font, in the selected FLC mixtures determined during heating.

Mixture Acronym	Cr	▪	SmC*	▪	SmA*	▪	N*	▪	Iso
A-MchM	▪	4.2 *11.74*	▪	70.2 ^#^ -	▪	73.6 ^#^ -	▪	74.5 *5.58*	▪
A-BchM	▪	−12.6 *7.28*	▪	64.3 ^#^ -	▪	73.5 *4.89*	-	-	▪
CchM	▪	−24.1 *1.88*	▪	62.9 ^#^ -	▪	65.2 *3.11*	-	-	▪

^#^—the phase transition temperatures were detected by POM as the DSC peak corresponding to this phase transition is too small.

**Table 6:** Temperature range of ferroelectric phase and collected electro-optical and physicochemical properties for investigated mixtures and top-modern NFLC-4-eut [44] mixture at T = 30 °C.

Mixture Acronym	Temperature Range of SmC* [°C]	Properties at 30 °C				
		*p* [nm]	*θ* [°]	*P_S_* [nC/cm^2^]	*τ* [μs]	*γ_φ_* [Pa·s]
A-MchM	4.2 < SmC* > 74.5	>2000	34.0	11	790	0.67
A-BchM	−12.6 < SmC* > 82.4	160	34.5	255	156	3.04
CchM	−24.1 < SmC* > 67.2	<240	36.0	217	362	6.00
NFLC-4-eut [44]	18.0 < SmC* > 60.0	~150	~30.0	~75	200	0.10

## Data Availability

The original contributions presented in this study are included in the article/Supplementary Material. Further inquiries can be directed to the corresponding author.

## Supplementary Materials

The following supporting information can be downloaded at: https://www.mdpi.com/article/10.3390/ma18102343/s1. Table S1. The phase transition temperatures [°C] form POM (first row) ad DSC (second row), and corresponding enthalpy changes [kJ mol^−1^], in italic font, of all prepared mixtures determined during heating; Figure S1. The DSC tracers of mixture A-MchM (a), A-BchM (b) and CchM (c) in the heating cycle (down curve) and cooling cycle (upper curve); Figure S2. Spectra of the A-BchM mixture at different temperatures, with arrows indicating weak full-pitch bands from selective reflection of the helical structure.
