# Supplementary material for: Ferroelectric Smectic Liquid Crystalline Materials with Different Degree of Chirality
Source: Materials (Basel). 2025 May 17;18(10):2343. doi: 10.3390/ma18102343 (PMC12113150; doi:10.3390/ma18102343)
Supplement: Supplementary file 1 [file materials-18-02343-s001.zip › materials-3615425-supplementary.pdf]

Supplementary Material

Table S1. The phase transition temperatures [°C] form POM (first row) ad DSC (second row), and corresponding enthalpy changes [kJ mol^{-1}], in italic font, of all prepared mixtures determined during heating.

Mixture Acronym	Cr	▪	SmC*	▪	SmA*	▪	N*	▪	Iso
AchM	▪	-<br>7.9<br>*14.07*	▪	68.5<br>69.81<br>*0.0089*	▪	83.2<br>81.28<br>*1.79*	▪	86.3<br>85.55<br>*3.25*	▪
0,2 MchM<br>0,8 AchM	▪	-<br>5.0<br>*14.87*	▪	72.1<br><br>*0.0166*	▪	78.4<br>78.3<br>*1.88*	▪	81.5<br>81.4<br>*2.62*	▪
0,4 MchM<br>0,6 AchM<br>**(A-MchM)**	▪	-<br>4.2<br>*11.74*	▪	70.2<br>-<br>-	▪	73.6<br>74.5<br>*5.58*	▪	76.1<br>-<br>-	▪
0,5 MchM<br>0,5 AchM	▪	-<br>-<br>-	▪	68.0<br>-<br>-	▪	70.3<br>-<br>-	-	-	▪
0,6 MchM<br>0,4 AchM	▪	-<br>-<br>-	▪	65.8<br>-<br>-	▪	67.8<br>-<br>-	-	-	▪
0,8 MchM<br>0,2 AchM	▪	-<br>5.0<br>*19.47*	▪	70.9<br>-<br>-	-	-<br>78.3<br>*2.74*	-	-<br>81.4<br>*3.71*	▪
MchM	▪	44.5<br>45.6<br>*12.59*	▪	79.4<br>80.5<br>*4.24*	-	-	-	-	▪
0,95 AchM<br>0,05 BchM	▪	-<br>6.1<br>*11.96*	▪	70.2<br>-<br>-	▪	83.3<br>84.1<br>*2.13*	▪	84.9<br>85.8<br>*2.80*	▪
0,8 AchM<br>0,2 BchM	▪	-<br>0.8<br>*10.45*	▪	74.6<br>-<br>-	▪	80.3<br>82.4<br>*5.14*	-	-	▪
0,6 AchM<br>0,4 BchM	▪	-<br>-12.6	▪	64.3<br>-	▪	71.3<br>73.5	-	-	▪

**(A-BchM)**		*7.28*		-		*4.89*			
BchM	▪	63.4<br>62.4<br>*20.36*	-	-<br>-<br>-	-	-	-	-	▪
0,95 A-MchM<br>0,05 BchM	▪	-<br>1.3<br>*12.95*	▪	74.6<br>74.6<br>*5.60*	-	-	-	-	▪
0,9 A-MchM<br>0,1 BchM	▪	-<br>-0.5<br>*9.66*	▪	73.5<br>74.7<br>*5.17*	-	-	-	-	▪
0,8 A-MchM<br>0,2 BchM	▪	-<br>-2.5<br>*3.88*	▪	72.7<br>-<br>-	▪	74.7<br>74.6<br>*2.38*	-	-	▪
0,6 A-MchM<br>0,4 BchM<br>**(CchM)**	▪	-<br>-24.1<br>*2.24*	▪	62.9<br>-<br>-	▪	64<br>65.2<br>*3.73*	-	-	▪
0,4 A-MchM<br>0,6 BchM	▪	-<br><-30.0<br>-	▪	50.9<br>47<br>*1.99*	▪	52.6<br>54.1<br>*0.34*	-	-	▪

a)

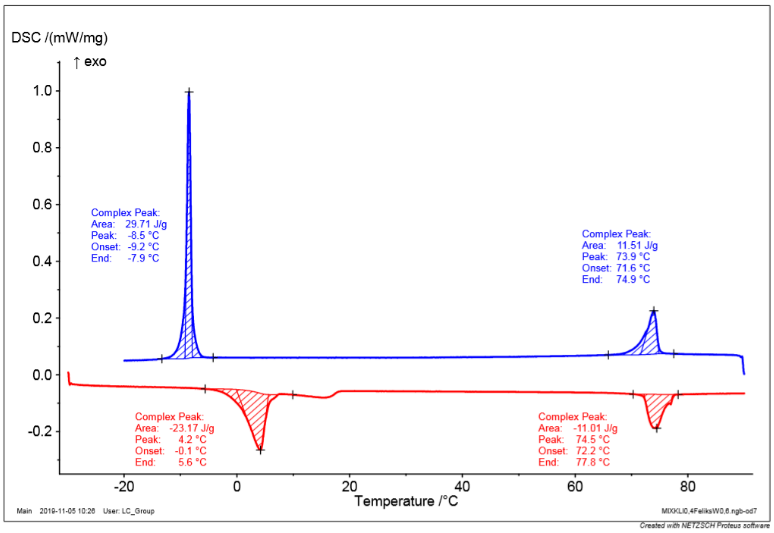

b)

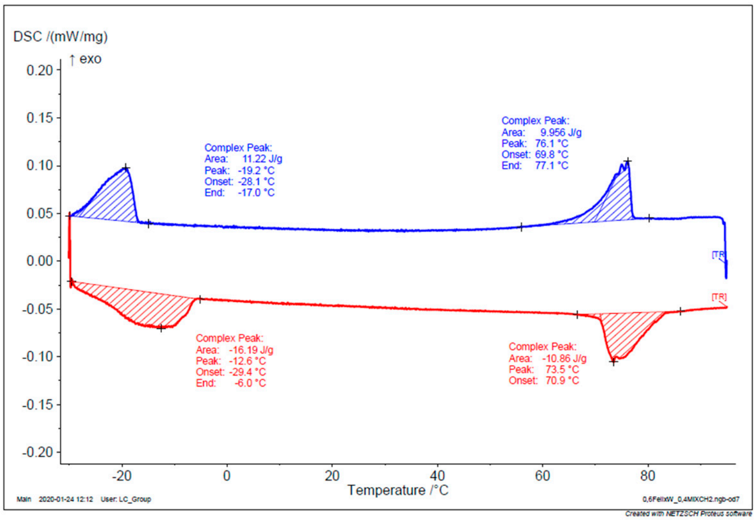

c)

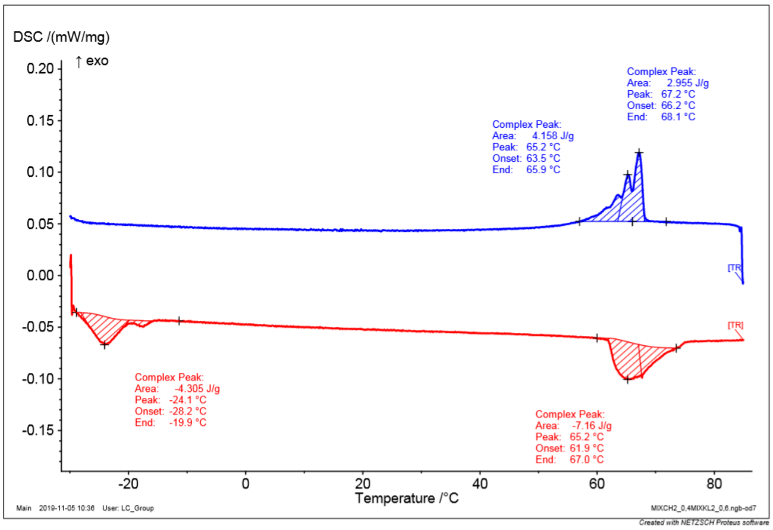

Figure S1. The DSC tracers of mixture A-MchM (a), A-BchM (b) and CchM (c) in the heating cycle (down curve) and cooling cycle (upper curve).

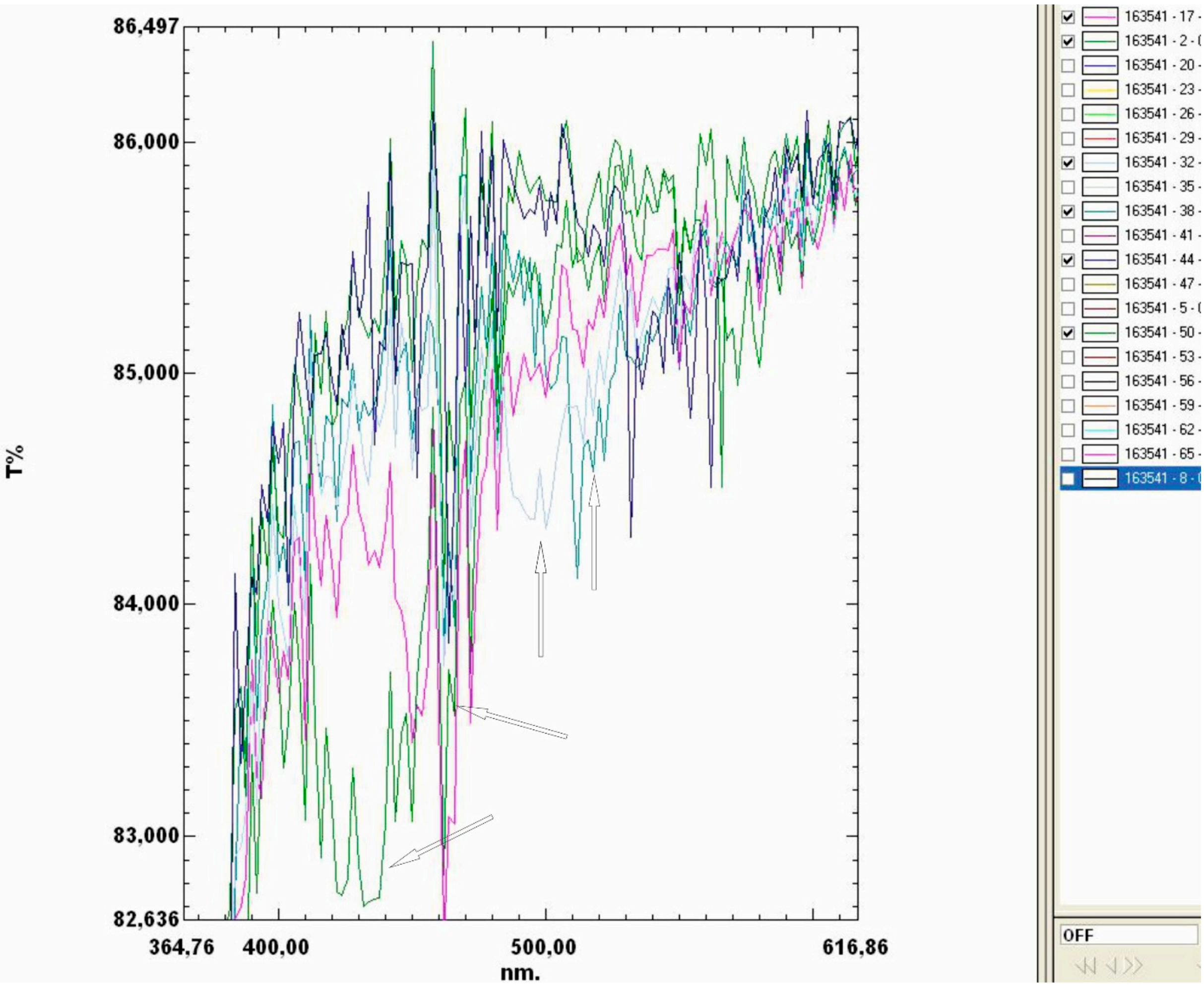

Figure S2. Spectra of the A-BchM mixture at different temperatures, with arrows indicating weak full-pitch bands from selective reflection of the helical structure.
